# The role of IL-37 in gastrointestinal diseases

**DOI:** 10.3389/fimmu.2024.1431495

**Published:** 2024-08-14

**Authors:** Qiang Wang, Guangrun Zhang, Caiping An, Brett D. Hambly, Shisan Bao

**Affiliations:** ^1^ Department of Anatomy, School of Basic Medicine, Gansu University of Chinese Medicine, Lanzhou, China; ^2^ School of Traditional Chinese and Western Medicine, Gansu University of Chinese Medicine, Lanzhou, China; ^3^ Department of Nephrology, Gansu Provincial Hospital, Lanzhou, China; ^4^ Centre for Healthy Futures, Torrens University Australia, Sydney, NSW, Australia; ^5^ Foreign Affairs Office, The Third Affiliated Hospital of Gansu University of Chinese Medicine, Baiyin, China; ^6^ Foreign Affairs Office, The First People’s Hospital of Baiyin, Baiyin, China

**Keywords:** IL-37, gastrointestinal, inflammation, cancer, colorectal cancer

## Abstract

Gastrointestinal mucosal surface is frequently under challenge due to it’s the large surface area and most common entry of microbes. IL-37, an anti-inflammatory cytokine, regulates local and systemic host immunity. *H. pylori* infection leads to the inhibition of IL-37 in the gastric mucosa, contributing to heightened mucosal inflammation and destruction, thereby facilitating increased proliferation of *H. pylori*. Food allergy, due to immune dysregulation, also contribute to GI injury. On the other hand, elevated levels of IL-37 observed in gastric cancer patients align with reduced host immunity at the cellular and humoral levels, indicating that IL-37 may contribute to the development of gastric cancer *via* suppressing pro-inflammatory responses. While IL-37 provides protection in an IBD animal model, the detection of highly produced IL-37 in IBD patients suggests a stage-dependent role, being protective in acute inflammation but potentially exacerbates the development of IBD in chronic conditions. Moreover, elevated colonic IL-37 in CRC correlates with overall survival time and disease time, indicating a protective role for IL-37 in CRC. The differential regulation and expression of IL-37 between upper- and lower-GI organs may be attributed to variations in the microbial flora. This information suggests that IL-37 could be a potential therapeutic agent, depending on the stage and location.

## Introduction

### Intestinal mucosal immunity and GI defense

The gastrointestinal (GI) system, encompassing the largest mucosal surface area in the body (1), is highly susceptible to both specific and non-specific microbial invasion (2). Gut-associated lymphoid tissues (GALT) within the GI system protects against such threats, and consists of lamina propria lymphocytes, intraepithelial lymphocytes (3), in addition to mesenteric lymph nodes. Any compromise or hyperactivity in GALT immunity can lead to severe consequences. For instance, a deficiency in IFNγ compromises host intestinal immunity at the cellular and humoral levels by impairing macrophage function in response to *Salmonella* challenge, resulting in salmonellosis and septicemia (4). On the other hand, exogenous IFNγ has been shown to protect the host against lethal salmonellosis *in vivo* in mice (5). Conversely, TNF, a pro-inflammatory cytokine, plays a crucial protective role in acute inflammatory bowel disease within the GI tract. Studies indicate that a deficiency in TNF could exacerbate colitis in a DSS-induced colitis murine model (6), maybe *via* upregulating IL-1β production, which is supported by their testing, using bone marrow derived dendritic cells from TNF KO mice (7), highlighting the protective function of TNF during acute inflammation. On the other hand, TNF is significantly upregulated, both circulating and within local affected intestinal tissues, indicating its pro-inflammatory role during chronic intestinal inflammation, which is supported by clinical findings, showing that anti-TNF antibody therapy significantly improves the condition of IBD patients at both the macroscopic and microscopic levels (8). Furthermore, if chronic inflammation persists without proper management in the intestine, it may lead to the induction of malignancy (9). This underscores the delicate balance required to maintain optimal GI health, with both excessive and insufficient immune responses posing significant risks. This review focuses on the role of IL-37 in both upper and lower GI infection/inflammation, as well as malignancies, which could provide a useful perspective for both basic scientists and clinicians.

## IL-37

IL-37, also known as interleukin-1 family member 7 (IL-1F7), exists in five different splice variants (a-e) (10). The IL-37 protein ranges in size from 17 to 26 kDa, corresponding to a gene size of 3.617 kb (11). It has been identified in various tissues, including lymph nodes, thymus, lung, intestine, uterus, as well as in leucocytes such as NK cells, activated B cells, and monocytes. Additionally, IL-37 has been detected in epithelial cells, suggesting a potential role in regulating intestinal mucosal immunity (12). The involvement of IL-37 in host immunity has demonstrated that IL-37 acts as an anti-inflammatory cytokine by inhibiting pro-inflammatory responses (12), and is able to attenuate both innate (13) and adaptive immunity (14). Additionally, dysregulated expression of IL-37 has been observed in autoimmune diseases such as psoriasis, Graves’ disease, and systemic lupus erythematosus (15), highlighting its crucial role in maintaining host homeostasis. Additionally, inflammatory bowel disease (IBD) involves, in part, an effector T cell response against commensal microbiota, which contributes towards disease severity, since ongoing inflammation in IBD leads to the loss of tolerance toward commensals and subsequent worsened disease outcomes. Specifically, the intestinal mucosal immune response is dependent on the differentiation of pro-inflammatory commensal antigen-specific T cells (e.g. against CBir1-bacterium) prior to intestinal damage (16). Thus, the role of IL-37 in regulating gastrointestinal mucosal immunity may also depend on the local immune response to various stimuli, leading to diverse outcomes.

## IL-37 in *H. pylori* gastric infection/inflammation

The ground-breaking discovery of the critical role played by *H. pylori* infection in contributing to gastric ulceration, and subsequent gastric cancer was elegantly demonstrated by Nobel laureates Drs Marshall and Warren (17). This revelation revolutionized the management of *H. pylori* infected gastritis patients, leading to fundamental improvements in their outcomes. The Nobel Committee commented on the significance of this discovery, stating, “Thanks to the pioneering discovery by Marshall and Warren, peptic ulcer disease is no longer a chronic, frequently disabling condition but a disease that can be cured by a short regimen of antibiotics and acid secretion inhibitors” (18).

Thus, the first research focus has been on the relationship between *H. pylori* gastric infection and IL-37 expression. A notable decrease in IL-37 expression was identified in the ulcerated gastric mucosa of biopsy samples from patients with *H. pylori* infection, in comparison to those without *H. pylori* infection (19). Furthermore, diminished IL-37 levels were also noted in the mucosa affected by gastritis with *H. pylori* infection, even in the absence of stomach ulceration (19). Surprisingly, no significant disparity in suppressed mucosal IL-37 levels was observed between *H. pylori*-infected patients with and without stomach ulceration.

These data imply that *H. pylori* promotes local inflammation *via* inhibiting the production of IL-37 in gastric mucosa, which in turn enhances the production of pro-inflammatory cytokines and chemokines (13), such as IFN-γ and TNF (20). Although pro-inflammatory responses provide defense, if the inflammation persists too long, particularly among susceptible individuals without proper management, local inflammation may not be sufficiently efficient to eradicate *H. pylori* infection, but rather will contribute to more severe disturbance in the stomach mucosa, resulting in persistent chronic infection. The severely inflamed tissues in the stomach mucosa may not then respond well to either pro and/or anti-inflammatory cytokine/chemokine signaling, which may promote further *H. pylori* infection. Thus, as stated above, these data highlight the role of pro-inflammatory mediators (e.g., IFN-γ (4) and TNF (6)) in providing protection against the severity of pathogenic challenges in the gastrointestinal mucosa, highlighting the pivotal role of pro-inflammatory mediators in preventing overwhelming inflammation.

The source of gastric mucosal IL-37 production remains unclear, but IL-37 is likely produced by immune and non-immune cells, including epithelial cells (21) in response to *H. pylori* challenge in an autocrine and paracrine fashion.

The discovery of a reduction in IL-37 in stomach tissue infected with *H. pylori in vivo* is consistent with findings *in vitro* (20), showing that reduction in mucosal IL-37 in the microenvironment may contribute to elevated levels of pro-inflammatory mediators, including chemokines, *in vivo*, and also in challenged GI epithelial cells *in vitro* (22). This is supported by findings indicating substantial IL-37-modulated chemokine production *in vitro* by GI epithelial cells, potentially leading to the persistence of chronic inflammation in the stomach. There are consistent findings of the function of IL-37 between *in vivo* and *in vitro* systems, supporting the view that gastric mucosal IL-37 might contribute to chronicity *via* suppressing local inflammation/immunity.

Overall, the suppressed IL-37 levels observed in patients with *H. pylori* infection may lead to a decrease in local anti-inflammatory responses and an increase in maladaptive damaging pro-inflammatory responses. While an enhanced pro-inflammatory response is typically crucial for defense against *H. pylori* infection, susceptible individuals may experience a disturbance in their host immunity. This disturbance could result in severe ulceration and inflammation of the gastric mucosa, potentially further promoting *H. pylori* infection and progressing to precancerous and/or gastric cancer stages. This aligns with the notion that chronic inflammation plays a critical role in the development of gastric cancer in infected individuals (23), and in some cases, a smaller number of patients may even develop extra-gastric mucosa-associated lymphoid tissue (MALT) lymphoma (24).

## Food allergy and coeliac disease

Food allergy and coeliac disease still lead to substantial morbidity in humans, despite extensive clinical and basic research over the last few decades (25). Following an extensive literature search, no published data on the role of IL-37 in food allergy or coeliac disease has been found. However, it has been reported that IL-37 is substantially reduced in asthmatic children (26), suggesting the important role of IL-37 in asthma. Additionally, exogenous IL-37 ameliorates allergic inflammation in asthmatic animal models, by suppressing pro-inflammatory cytokines, e.g. IL-1 and IL-33 (27), which is supported by findings in IL-37 transgenic mice. Considering that both intestinal and respiratory mucosal surfaces belong to mucosal-associated lymphoid tissues (MALT) (28), it is reasonable to speculate that IL-37, acting as an anti-inflammatory cytokine, could inhibit food allergy by upregulating CD35^+^ Treg cells (29), thereby dampening hyperactive MALT responses (30). Consequently, IL-37 could serve as a therapeutic target for managing food allergy, similar to the role described for IL-33 (31), which requires verification in future studies. The precise underlying mechanism of IL-37 in regulating allergic response remains to be clarified. A recent report finds that IL-37 ameliorates local inflammation in atopic dermatitis by regulating gut microbiota through the AMPK-mTOR signaling pathway (32), further supporting the concept that IL-37 may act on both local and systemic responses *via* manipulating intestinal mucosal microbiota, an idea that requires further investigation.

Coeliac disease, an autoimmune disorder primarily affecting the small intestine, results from the ingestion of gluten-containing foods by genetically susceptible individuals with specific HLA alleles (33). Histopathological examination of coeliac disease reveals varying degrees of mucosal inflammation, characterized by cellular and humoral immune responses, including leukocyte infiltration, swelling, and neovascularization (34). Elevated levels of pro-inflammatory cytokines and reports of anti-inflammatory cytokines in coeliac disease patients correlate with clinical severity (35), suggesting a close association between coeliac disease and heightened pro-inflammatory cytokine production. However, attempts to mitigate inflammation in this setting, through anti-inflammatory cytokines, may be insufficiently effective. Recent studies have clarified the gluten-specific host response, demonstrating a strong correlation between gluten intake and clinical symptoms and signs in coeliac patients compared to controls (36). This observation aligns with significantly increased levels of pro-inflammatory cytokines, such as IL-2, IL-6, CCL20, CXCL8, CXCL9, IFN-γ, alongside elevated anti-inflammatory cytokines including IL-10 and IL-22. Because there is no direct evidence of the relationship between IL-37 and coeliac disease, based on the above data, it is also reasonable to speculate that IL-37, acting as an anti-inflammatory cytokine, would be enhanced among coeliac disease patients, particularly acutely following the ingestion of gluten contained food. This speculation is purely based on indirect evidence and consequently requires further verification.

## IL-37 in gastric cancer

Persistent chronic stomach ulceration, particularly in the presence of *H. pylori* infection, substantially boosts the incidence of gastric cancer (37). Notably, circulating IL-37 is significantly higher in gastric cancer patients, compared to sex and age matched healthy controls (38), suggesting a possible pro-tumor effect of IL-37, since elevated IL-37 promotes an anti-inflammatory response *via* suppressing both innate (13) and adaptive immunity (14), resulting in compromised host immune surveillance against malignancy (39). These data are supported by the finding that IL-37 inhibits the immune response at both the cellular and humoral levels (40), which likely promotes gastric cancer development *via* inhibiting host gastric mucosal immunity against the development of gastric cancer. These data are further consistent with the finding that there is an inverse correlation between circulating IL-37 expression and 5-year survival (38). The pro-cancer role of IL-37 in gastric cancer is further sustained from multivariate analysis, which provides a more reliable prediction than univariate, showing that while the depth of invasion (T1-2 *vs* T3-4) and stage (I-II *vs* III-IV) were significant under univariate analysis, IL-37 expression (low *vs* high) remained significant under multivariate analysis and was the most reliable predictor for overall survival and progression free survival using multi-variate analysis. Interestingly, only high circulating IL-37 expression is a reliable predictive factor for low progression-free survival. Additionally, an elevated level of circulating IL-37 was correlating with poor differentiation. Surprisingly, there is no evidence in the literature about the specific underlying mechanism concerning the pro-cancer activity of IL-37 in gastric cancer, which warrants further investigation. There is at least a partial explanation that IL-37 inhibits immunosurveillance, although these data are not specific for gastric cancer, they are a more general immunological observation (13, 39–41). The underlying mechanism in gastric cancer has not been specifically examined.

Interestingly, in most other cancers examined to date, elevated levels of IL-37 have correlated with anti-tumor activity and with improved survival (12). IL-37 has been shown to be protective during the development of a number of cancers, including hepatocellular carcinoma (42, 43), colorectal cancer (44), non-small cell lung cancer (45), renal cell carcinoma and oral and cervical squamous cell carcinoma (46). Possible anti-tumor mechanisms of IL-37 include inhibition of both angiogenesis and tumor-promoting inflammation, and promotion of anti-tumor immunity (47). Paradoxically, high circulating levels of IL-37 have been shown to associated with decreased survival in patients with metastatic epithelial ovarian cancer (48).

However, the detailed data within the literature are very variable, as some studies investigated circulating IL-37, while others have measured cancer vs normal tissue levels of IL-37. Data derived from other cancers suggest that the best indicator of IL-37 activity is likely to be the level of the IL-37 in cancer tissue as a function of severity/survival.

Such speculation is aligned with our previous findings, showing that there is an inverse correlation between IL-31, -32, -33 (49) and IL-34 (50) in gastric cancer survival, suggesting a pro-cancer role of these cytokines. Taken together, these data align with the concept that host immunity plays a critical role in immune surveillance against the development of gastric cancer, i.e. both mucosal and systemic immunity may be compromised among gastric cancer patients due to significantly upregulated IL-37, which inhibits potentially protective pro-inflammatory responses (51).

Interestingly, a recent report shows that IL-37 can play a dual role in malignancy, i.e. IL-37 possesses both anti-inflammatory and pro-inflammatory functions (52), depending on which subset units of IL-37 are activated. A protective role of IL-37 during the development of gastric cancer has been demonstrated, where the processed form of IL-37 binds to SMAD-3, relocates to the nucleus, and hinders the transcription of various pro-inflammatory genes. Both the precursor and cleaved versions of IL-37 are actively secreted. They associate with the IL-18Rα chain, shared by IL-18 as a receptor subunit, and recruit Toll/IL-1R (TIR)-8 to facilitate intracellular signaling. IL-37 suppresses the activation of NF-κB and MAPK while activating the Mer-PTEN-DOK pathway. It exerts negative regulation on signaling induced by TLR agonists, proinflammatory cytokines, and IL-1RF ligands. Additionally, IL-37 influences cell metabolism by inhibiting mTOR and GSK-3α/β, and activating AMPK (53).

By contrast to gastric cancer, it has been reported that IL-37 suppresses hepatocellular carcinoma (HCC) growth through inhibiting tumor-associated macrophages (TAM) (54), by promoting TAM polarization from the pro-tumorigenic M2 subtype to the anti-tumorigenic M1 subtype (55). A possible explanation for this discrepancy is that the exposure of the epithelial cells of the liver and stomach to microbiological flora are completely different, although both belong to the GI system. Thus, GI mucosal immunity may play different regulatory roles in maintaining homeostasis in these two different organs. This is in line with a report showing that the anti-inflammatory role of IL-37 contributes to the suppression of chronic inflammation, particularly among patients with non-alcoholic fatty liver disease (NAFLD), and subsequently reduces the risk of malignancy (56).

Surprisingly, no studies have examined local gastric mucosal expression of IL-37 amongst either GC patients or HCC up to date. It is understandable that there are ethical challenges to obtaining gastric mucosa or hepatic tissue from HCC cohorts, but certainly the detection of IL-37 in GC tumor and adjacent normal gastric mucosa warrants further clarification. Importantly, recent research indicates that IL-37 could serve as a novel therapeutic tool for cancer patients (53), and there is growing evidence suggesting its potential role as a prognostic marker across various human cancers (56).

## IL-37 in IBD

Inflammatory bowel disease (IBD) serves as an umbrella term encompassing chronic gastrointestinal inflammation, which includes conditions such as ulcerative colitis and Crohn’s Disease (57). The etiology of IBD is intricate, involving factors such as environmental influences, genetics, and infections (57). The global incidence of IBD is 6.8 million (58). Notably, IBD is a lifelong condition currently lacking a cure, making it a significant focus of attention (58).

The protective role IL-37 during the development of experimental colitis has been demonstrated in IL-37 transgenic mice following DSS challenge, showing substantially reduced clinical signs and symptoms and histopathology severity from IL-37 overexpression transgenic mice, compared to that of wildtype counterparts (59). Importantly the induced IL-37 mRNA inversely correlates with intestinal barrier breakdown (59). The reduced intestinal mucosal inflammation in the IL-37 transgenic mice was found to be consistent with suppressed pro-inflammatory mediators (TNF and IL-1β, IL-17, IL-6 and CXCL1), but enhanced anti-inflammatory mediator (IL-10) production, and reduced recruitment of leucocytes (neutrophils, dendritic cells, macrophages, eosinophils) in the lamina propria (59). It has been elegantly demonstrated that hemopoietic-derived IL-37 provides an essential protective role from DSS colitis, by adoptive transfer of bone marrow from IL-37 transgenic mice, compared to that of the control bone marrow. Additionally, correlating with improved histopathology, increased Ki67 demonstrated proliferation and regeneration of intestinal mucosa. Furthermore, there was no significant difference in the group receiving anti-IL-10 receptor blocking antibody, possibly because IL-10 did not exhibit a syngeneic role with IL-37. Alternatively, the antibody may not have maintained a therapeutic effect for sufficient time.

Interestingly, conflicting results have emerged from IL-37 transgenic mice following DSS stimulation, revealing that IL-37 transgenic mice exhibited more severe colitis under conventional conditions compared to wildtype mice (60). Conversely, under SPF conditions, IL-37 transgenic mice displayed less severe colitis. This implies that the protective role of IL-37 is contingent upon the gut microbiota, specifically whether dysbiosis is present. This observation suggests a potential link between environmental factors and the pathogenesis of colitis, in conjunction with the integrity of the intestinal mucosal epithelial barrier. This barrier plays a pivotal role in recruiting neutrophils and NK cells, as well as preventing the invasion of pathogenic bacteria into the colon lamina propria and its draining lymph nodes (16), because intestinal damage is required for the pro-inflammatory differentiation of commensal antigen-specific T cells, for example against the CBir1-bacterium.

Therefore, the roles of intestinal mucosal IL-37, influenced by the state of the intestinal microbiota, may contribute to either the exacerbation or alleviation of IBD occurrences. The regulation of host cellular and/or humoral immunities based on gut pathogenic bacteria, or the maintenance of intestinal microbial and immune homeostasis, emerges as a promising therapeutic strategy for IBD (60).

The findings in the animal model of IBD are aligned with observations in patients with IBD, showing that significantly higher expression of IL-37β is observed in biopsies from inflamed mucosa from active ulcerative colitis patient (61), but there is no obvious expression of IL-37β in the normal intestinal mucosa. The upregulation of IL-37β is consistent with and likely a consequence of a high level of proinflammatory mediators, particularly TNF (61). These findings indicate that heightened levels of intestinal mucosal IL-37β in individuals with ulcerative colitis may play a protective or immunosuppressive role in response to local inflammation. However, persistent chronic inflammation in susceptible cohorts, potentially due to either inadequate activation of downstream pathways and/or ineffective receptors for IL-37 (61), compromise IL-37 action and subsequently disrupt the local immunological balance.

Eventually irreversible permanent damage occurs within the intestinal mucosa, despite the application of different approaches, e.g. NSAIDs, steroids, the anti-TNF monoclonal antibody biologics (infliximab, golimumab, certolizumab and adalimumab, and the fusion protein etanercept). There is no literature concerning the involvement of other subsets of IL-37 in IBD, which warrants further exploration. In particular, the role of specific subsets of IL-37, i.e. IL-37α and γ in patients with IBD remains to be explored in future.

The crucial role of IL-37 in the development of IBD is highlighted by findings in a pediatric patient (62), who developed infantile ulcerative colitis, as a consequence of the over-expression of an inactivated homozygous IL-37 variant that was functionally unable to suppress pro-inflammatory cytokine production. The patient’s mother was identified as heterozygous for the inactivating IL-37 variant mutation. This genetic variant may contribute to the destabilization of the protein structure of IL-37, leading to increased solvent accessibility of the substituted polar residue (62). These clinical findings offer insights into the role of IL-37 during IBD development and suggest it could be a potential therapeutic target for managing patients with IBD in the future.

Further mechanistic investigation from this study illustrates that the mutant Ile177Thr IL-37 exhibits lower stability than wild-type IL-37, rendering the mutant IL-37 more susceptible to eradication, determined using cycloheximide chase assays, despite the elevated protein expression of I177T IL-37 (62). This insight is supported by *in vitro* studies, demonstrating that primary monocyte-derived dendritic cells from this IL-37 variant patient produce higher levels of TNF and IFN-γ compared to wild-type cells. Furthermore, these cells are unable to effectively inhibit pro-inflammatory responses *in vitro*, confirming the underlying mechanism of the IL-37 variant in response to pro-inflammatory stimulation.

It is important to highlight the substantial differences between IBD in humans and animal models. For instance, the duration of IBD often exceeds 10 years in humans, whereas in animal models, it typically spans only a few weeks. Consequently, there are significant variations in host immunity between chronic and acute conditions.

## IL-37 in colorectal cancer

Colorectal cancer (CRC) remains a formidable global health challenge, despite extensive research efforts spanning both basic and clinical domains over several decades (63). CRC is still ranked as one of the leading prevalent malignancies worldwide, with an annual incidence of 1.93 million new cases (64). Effectively managing CRC continues to prove to be a significant struggle for clinicians and patients alike. Consequently, patients often experience unfavorable outcomes and a low five-year survival rate (65), primarily due to a substantial proportion being diagnosed at advanced stages characterized by deep bowel wall invasion and/or distant metastasis, with palliative care being the only option for these patients. It is well known that host immunity plays a critical role in tumorigenesis, especially in CRC, consistent with reports showing that there is a positive correlation between pro-inflammatory mediators and the severity of CRC (66), especially in the majority of CRC that are associated with MMR/MSI incompetent tumors (67).

The precise role of IL-37, an anti-inflammatory cytokine, during the development of CRC remains to be explored in detail. However, it has been hypothesized that IL-37 may provide protection *via* regulating host MALT immunity during the development of CRC, based partly on the observation that colonic IL-37 is significantly diminished in CRC tissues, evident at both the mRNA and protein levels, when compared to non-cancerous tissues (66).

The proposed protective role of IL-37 in CRC is supported by the observation that an inverse correlation occurs between colonic IL-37 and CRC invasion and differentiation (66). Additionally, a positive correlation occurs between IL-37 expression levels and both disease-free survival and overall survival (66), further supporting our hypothesis. Another study has revealed that IL-37 is localized in the cytoplasm of colonic epithelial cells, and the expression of colonic IL-37 in CRC tissue is consistently reduced compared to that in non-CRC colonic epithelial cells (68). Notably, in this study, colonic tumor IL-37 expression exhibits an inverse correlation with the depth of CRC invasion, consistent with CRC progression (68). Surprisingly, no significant correlation was observed between colonic tumor IL-37 expression and differentiation of CRC by the second research team (68). This discrepancy, i.e. the correlation between IL-37 and differentiation of CRC, reported by these two groups, may be due to different patient demographics, such as different regional, genetic and/or environmental backgrounds, which should be further verified. No differences have been observed in the role and expression of IL-37 in CRC as a function of the location of the CRC (right *vs* left colon) (69), or patient sex (70) or age (71).

In contrast to the aforementioned findings, increased IL-37 levels appear to promote CRC tumorigenesis in the context of chronic inflammatory bowel disease, revealing a more intricate mechanism (72). To investigate how IL-37 contributes to CRC promotion, researchers utilized IL-37 transgenic mice to induce CRC in the presence of chemically-induced colitis (72). Compared to wild-type (WT) mice with colitis, the IL-37 over-expression transgenic mice showed more severe colitis and a greater number of tumors. Notably, IL-37 transgenic mice exhibited compromised CD8^+^ T cell function, leading to enhanced evasion of immune surveillance. Moreover, dysfunctional CD8^+^ T cells mediated IL-37’s inhibition of IL-18-induced proliferation and effector function, a process dependent on SIGIRR (single immunoglobulin interleukin-1 receptor-related protein) (72). These findings underscore that the overall impact of IL-37 expression heavily relies on the pre-existing inflammatory status of the mucosa.

**Figure 1:**
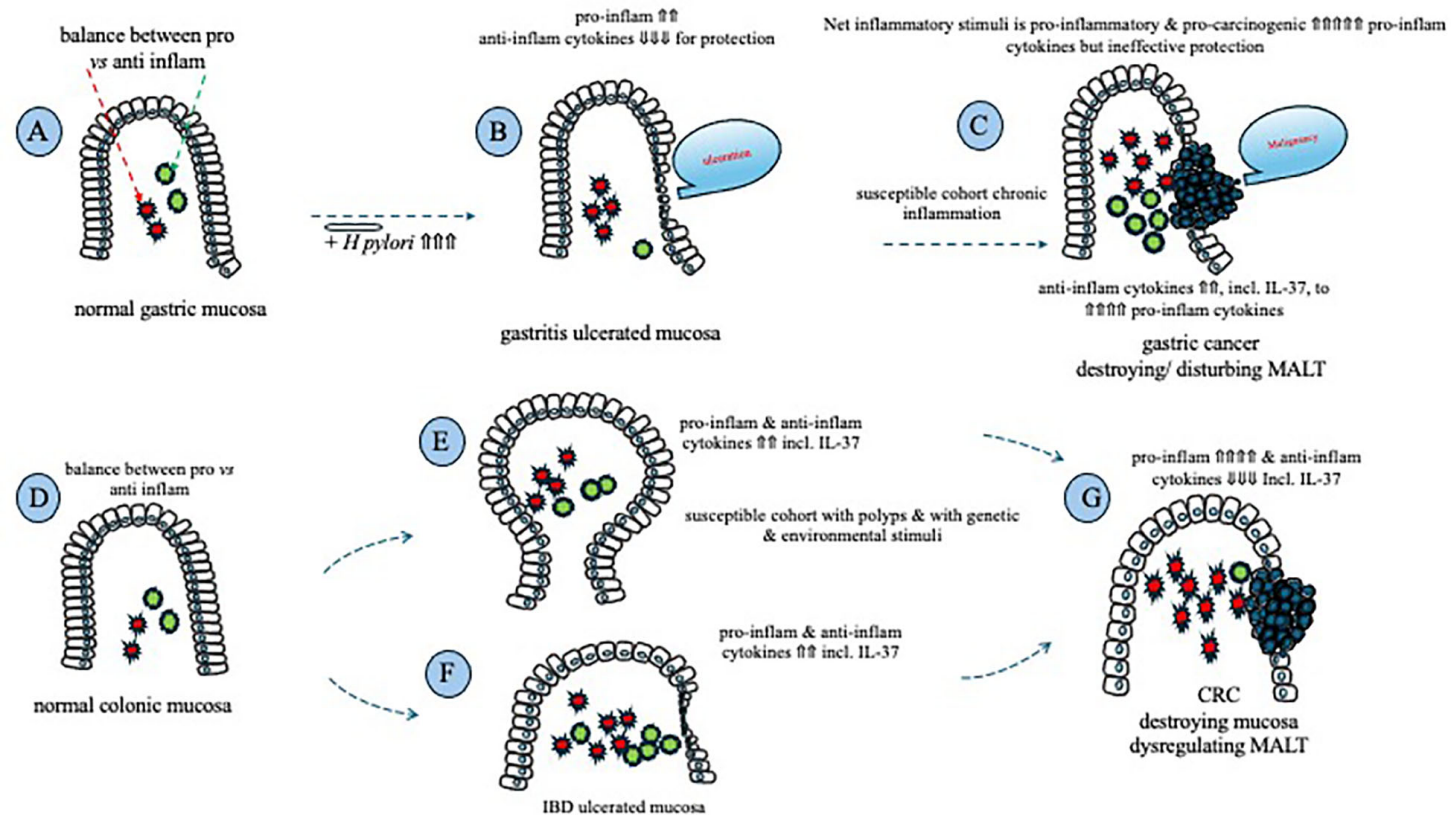
Schematic that summarizes the involvement of inflammatory cytokines in mucosal ulceration and cancer formation in the stomach and the colon. Homeostasis between pro- and anti-inflammatory cytokines exists within the normal gastric mucosa **(A)**; chronic *H. pylori* infection results in an increase in pro-inflammatory cytokines, with a reduction in anti-inflammatory cytokines, including IL-37, leading to gastric ulceration **(B)**; following prolonged ulceration, anti-inflammatory cytokine expression rises, but is unable to suppress a continued rise in pro-inflammatory/pro-carcinogenic cytokines, ultimately leading to gastric cancer **(C)**. Homeostasis between pro- and anti-inflammatory cytokines exists within the normal colonic mucosa **(D)**; both pro- and anti-inflammatory mucosal cytokines are increased within a cancer-susceptible cohort **(E)**, +/- the presence of IBD **(F)**, in an attempt to maintain homeostasis; prolonged pro-inflammatory/pro-carcinogenic cytokine stimulation leads to neoplastic transformation of colonic mucosal epithelial cells that reduce their production of anti-inflammatory cytokines, including IL-37, resulting in the formation of CRC **(G)**.

At the molecular and signaling levels, IL-37, recognized as an anti-inflammatory cytokine (13), plays a critical role in maintaining the integrity of intestinal mucosal homeostasis. When the mucosa faces various pathological stimuli or challenges, including exacerbations of IBD, colonic IL-37 is released to alleviate inflammation (73). This process can help prevent the transformation of colonic epithelial cells into a malignancy by suppressing the pro-proliferative stimulus of pro-inflammatory cytokines. However, if the underlying pathological stimuli are not effectively addressed or halted, chronic inflammation persists, for example, chronic inflammation associated with IBD, disrupting tissue homeostasis (67), despite ongoing efforts by colonic IL-37 to suppress local inflammation. As mentioned earlier, immune surveillance against malignancies relies on host immunity and inflammation; therefore, prolonged and excessive suppression of pro-inflammatory responses may potentially promote tumorigenesis by hindering the local inflammation necessary for tumor cell destruction (74). This aligns with previous research demonstrating a positive correlation between pro-inflammatory mediators and CRC histopathology (67) (**Figure 1**).

## Comparison between up and low GI cancers

Moreover, the regulatory role of IL-37 varies between gastric cancer and colorectal cancer, promoting carcinogenesis through the up-regulation of IL-37 in gastric cancer or the down-regulation of IL-37 expression in colorectal cancer within the local mucosa. This difference can be attributed to the substantial variations between these two gastrointestinal organs. A significant factor is the distinction in the microenvironments of the colon and stomach, despite both being part of the MALT system (28). In addition, genetic contributions to tumorigenesis in both gastric cancer and colorectal cancer, local microenvironments also play a role in carcinogenesis, such as *H. pylori* in gastric cancer (37) and ultra-processed rich food in colorectal cancer (75). These differences necessitate distinct host mucosal regulatory responses to maintain homeostasis. Consequently, the signaling pathways for gastric cancer and colon cancer differ, suggesting the induction of different immune-regulatory mechanisms, particularly on IL-37 during tumorigenesis.

The above observations invite speculation that development of gastric cancer and CRC is partially due to substantially impaired/disturbed local and possible systemic host immunity among susceptible individuals, for example intestinal mucosal IL-37 production. Dramatic impaired host immunity compromised immune surveillance against the development of malignancy, resulting in initiation of cancers (51).

Interestingly, IL-37 has been shown to exert protective effects in various cancer types. For instance, in hepatocellular carcinoma (76), it may operate through the inhibition of M2 macrophages (54). In lung cancer (77), IL-37 demonstrates protective effects by inhibiting angiogenesis, as illustrated in an animal model study (78). From a mechanistic standpoint, elevated levels of IL-37 have been associated with increased infiltration of CD1a^+^ dendritic cells, which notably correlates with the overall survival rate in hepatocellular carcinoma (79). The implications of enhanced dendritic cells in anti-tumor immunity may involve heightened professional antigen presentation, leading to a subsequent increase in the differential polarization of macrophages (79). This phenomenon could elucidate the varying roles of macrophages in the development of malignancies, potentially exhibiting diverse mechanisms of carcinogenesis or microenvironments. The differential role of IL-37 in the GI tract and among different cancers may be due to the host differential immunological response(s) to various challenges, with different outcomes.

## Conclusion

*H. pylori* infection leads to the inhibition of IL-37 in the gastric mucosa, contributing to heightened mucosal inflammation and destruction, thereby facilitating increased proliferation of *H. pylori*. Elevated levels of IL-37 observed in gastric cancer patients align with reduced host immunity at the cellular and humoral levels, indicating that IL-37 may not play a protective role in gastric cancer. It is speculated that IL-37 may protect individuals from food allergy and/or coeliac disease. While IL-37 provides protection in an IBD animal model, the detection of highly produced IL-37 in IBD patients suggests a stage-dependent role, being protective in acute inflammation but potentially promoting IBD in chronic conditions. Moreover, elevated colonic IL-37 in CRC correlates with overall survival time and disease time, indicating a protective role for IL-37 in CRC, except possibly in IBD-associated CRC. The differential regulation and expression of IL-37 between upper- and lower-GI organs may be attributed to variations in the microbial flora. This information suggests that IL-37 could be a potential therapeutic agent, acting as a key suppressor of innate immunity and allergic immune responses mediated by leucocytes.
